# Evaluationof compact air-induction flat fan nozzles for herbicide applications: Spray drift and biological efficacy

**DOI:** 10.3389/fpls.2023.1018626

**Published:** 2023-02-03

**Authors:** Shilin Wang, Xinjie Li, David Nuyttens, Lanting Zhang, Yajia Liu, Xue Li

**Affiliations:** ^1^ College of Science, China Agricultural University, Beijing, China; ^2^ Institute of Agricultural Facilities and Equipment, Jiangsu Academy of Agricultural Sciences, Nanjing, Jiangsu, China; ^3^ Syngenta Nantong Crop Protection Co Ltd, Nantong, Jiangsu, China; ^4^ Flanders Research Institute for Agriculture, Fisheries and Food (ILVO), Merelbeke, Belgium

**Keywords:** nozzle, droplet size, deposition, drift potential, herbicide application

## Abstract

Nozzles are the most critical component of a sprayer for pesticide applications. Recently, air-induction nozzles and twin flat-fan air-induction nozzles have started to be used for herbicide applications. In order to evaluate the potential of compact air-induction nozzles for herbicide spraying, this paper compares the effects of air-induction nozzles and standard flat-fan nozzles on spray atomization, deposition, drift, and weed control efficacy in maize and wheat. Droplet spectra were measured by a laser particle size analyzer, and drift potential values were determined using a drift test bench (ISO 22401). A field study was conducted to compare the spray drift and biological efficacy between Lechler standard flat-fan nozzles and compact air-induction nozzles including different nozzle sizes. In the range from 0.2 to 0.4 MPa, the droplet size classes of the LU and ST nozzles were very similar and ranged from fine to very fine, while the droplets of the air-induction nozzles IDK and IDKT were medium or coarse depending on the spray pressure and nozzle size. The drift potential trials showed that the droplet size characteristics, mainly *V*_100_, are strongly linked with the drift reduction potential. Both drift potential and field results showed that the compact air-induction nozzles had a good performance in drift reduction. In terms of weed control biological efficacy, there were no significant differences between standard flat-fan nozzles and air-induction nozzles. In all cases, the efficacy values were above 80% both in maize and in wheat. In conclusion, air-induction nozzles are recommended for herbicide applications as they provide good biological efficacy while significantly reducing the amount of spray drift, which is of great significance for the protection of the environment and the surrounding sensitive crops.

## 1 Introduction

Effective spray deposition and drift reduction of herbicide applications have come to the forefront all over the world (Alves et al., 2017; Butts et al., 2019; Oliveira et al., 2019; Carbonari and Velini, 2021). A number of previous studies have shown that droplet size plays an important role on spray deposition and drift behavior (Ennis and Williamson, 1963; McKinlay et al., 1974; Lake, 1977; Knoche, 1994; Jensen et al., 2001; Wilkerson et al., 2004, Shan et al., 2021). In most cases, smaller spray droplets provide greater coverage and deposition of the target tissue, thus maximizing herbicide activity. Larger droplets have a higher kinetic energy and velocity (Nuyttens et al., 2009), which promote bouncing and shattering of the droplets on the leaf surface (Zwertvaegher et al., 2014; Delele et al., 2016; Franca et al., 2020). However, drift potential is closely related to droplet size. The smaller the droplet, the higher its drift potential (Hewitt, 1997; Miller and Tuck, 2005; Nuyttens et al., 2007; Stainier et al., 2006; Qin et al., 2010; Wang SL et al., 2018). For this purpose, it is of great significance to select the proper droplet size in the process of chemical weed control.

Nozzles are one of the most critical components of the sprayer for pesticide applications, which play a decisive role in the process of liquid atomization, transfer and impact on the targets, and deposition and drift characteristics of pesticide droplets (Butler-Ellis et al., 1997; Ferguson et al., 2015; Machado et al., 2019; Chen et al., 2022). According to the shape of the spray fan, nozzles can generally be divided into hollow cone and flat-fan nozzles. Meanwhile, each kind of nozzle can be subdivided into a variety of types, each one with their own droplet size characteristics—for example, the Lechler hollow cone TR, ITR, and TX nozzles and the Lechler flat-fan ST, LU, IDK, and IDKT nozzles. In practice, the nozzle should be selected based on the environmental conditions, the application target, and the types or formulations of the active ingredients (Al Heidary et al., 2014). In China, hollow cone nozzles are often used for fungicide and insecticide applications because of their acceptably uniform deposition in a large spray area, and flat-fan nozzles are the first choice for herbicide applications due to their superb uniformity of deposition.

Flat-fan nozzles produce a thin, flat liquid sheet spreading outwards from the nozzle tip. Based on flat-fan nozzles, air-induction (also called air injector or air inclusion) flat-fan nozzles have been developed to reduce spray drift, which was beneficial to target deposition (Vallet and Tinet, 2013). For air-induction nozzles, air is drawn into the liquid channel through aspiration holes and mixed with the spray liquid (Bai et al., 2013; Dacunha et al., 2020). Consequently, larger air-bubble-containing droplets are formed, which are scattered into smaller droplets when impacting on the target due to the splashing of the droplets (McArtney and Obermiller, 2008, Zwertvaegher et al., 2014; Delele et al., 2016). The use of air-induction nozzles is increasing in many European and American countries (Sousa Alves, et al., 2017).

Recently, some new compact anti-drift air-induction nozzles and twin flat-fan air-induction nozzles have started to be used for herbicide spraying (Jensen et al., 2001; Xie et al., 2020), such as the Lechler IDK and IDKT nozzles tested in this study. In recent years, more and more studies focused on the spraying performance of these newly designed nozzles and their efficacy using real Plant Protection Products (Creech et al., 2018; Dafsari et al., 2021; Gong et al., 2022). In order to highlight the role of the compact air-induction nozzle in herbicide spraying, this paper compares the spray performance of the compact air-induction nozzles with conventional flat-fan nozzles. The droplet spectra of the nozzles were measured and classified, and the linear relationship between the droplet size and drift reduction percentage was clarified. At the same time, the field drift and control efficacy of air-induction nozzles and conventional flat-fan nozzles were compared. All these aspects have an important guiding role in the appropriate selection of nozzles for the chemical control of weeds in the field.

## 2 Materials and methods

### 2.1 Spray application technologies

In the droplet size measurements and the drift potential trials, four nozzle types were evaluated: standard flat-fan ST and LU nozzles and compact air-induction flat-fan IDK and IDKT nozzles (Lechler GmbH, German). For the four nozzle types, droplet size spectra, spray deposition, and drift potential of both ISO 03 and ISO 05 nozzle sizes were tested at 0.2, 0.3, and 0.4 MPa. The corresponding flow rates are shown in **Table 1**.

**Table 1:** Flow rates (L min^-1^) of nozzles at different spray pressure values.

ISO nozzle size	Spray pressure (MPa)		
	0.2	0.3	0.4
03	0.97	1.19	1.37
05	1.61	1.97	2.28

The drift potential trials and field herbicide application with different types of nozzles were carried out with a 3WX-400 boom sprayer (manufactured by Beijing Fengmao Plant Protection Machinery Co., Ltd., China). The boom sprayer has a spray swath of 8 m, with a nozzle spacing of 0.5 m. Two experimental procedures were used in the herbicide application field trials, including the evaluation of spray drift deposition and biological efficacy of herbicides. In the field application, the boom height was 0.5 m above the ground, and the spray pressure was always 0.3 MPa.

In maize, flat-fan nozzles (LU120-03 and LU120-05) and compact air-induction nozzles (IDKT120-03 and IDKT120-05) were installed on the boom sprayer for the combined drift and pre-emergence herbicide application trials. The application plots were rectangular, with a size of 24 m × 100 m. There is a total of five operation plots: four plots were used for the herbicide application with each of the four application technologies and one for the non-treated control plot. A buffer zone with a width of 16 m (two swaths) was maintained between plots to avoid cross-contamination between treatments. During all applications, the travel speed of the boom sprayer was maintained at 4.0 km/h, resulting in application volumes of 348 and 590 L/ha, respectively, for the ISO 03 and ISO 05 nozzle sizes.

In winter wheat, flat-fan nozzles (LU120-03 and LU120-05) and compact air-induction nozzles (IDK120-03, IDK120-05, IDKT120-03, and IDKT120-05) were installed on the boom sprayer for post-emergence herbicide application. The application plots and buffer zones were similar as in the maize trial. There is a total of seven operation plots: six plots were used for the herbicide application with different nozzles and one was for the non-treated control plot. The application volume was 300 L/ha for each treatment. In order to ensure the same spray volume for the different types of nozzles, the travel speed of the sprayer with ISO 03- and ISO 05-sized nozzles were 4.8 and 7.9 km/h, respectively.

### 2.2 Droplet size measurements

The study was carried out at the Centre for Chemicals Application Technology of China Agricultural University. The droplet size spectra of different nozzle–pressure combinations were measured with a DP-2 laser particle size analyzer (Zhuhai OMEC Instruments Co., Ltd., China). The to-be-tested nozzles were fixed at 50 cm above the detection area of the laser particle size analyzer. Three replicate measurements were made for each nozzle–pressure combination, with each replication scanning the cross-sectional area of the spray for 10 s. The spray parameters of interest were volume median diameter (VMD) and the volumetric percentage of droplets smaller than 100 μm (*V*_100_), which are also referred to as driftable fine droplets (Sousa Alves et al., 2017).

### 2.3 Drift potential trials

**Figure 1:**
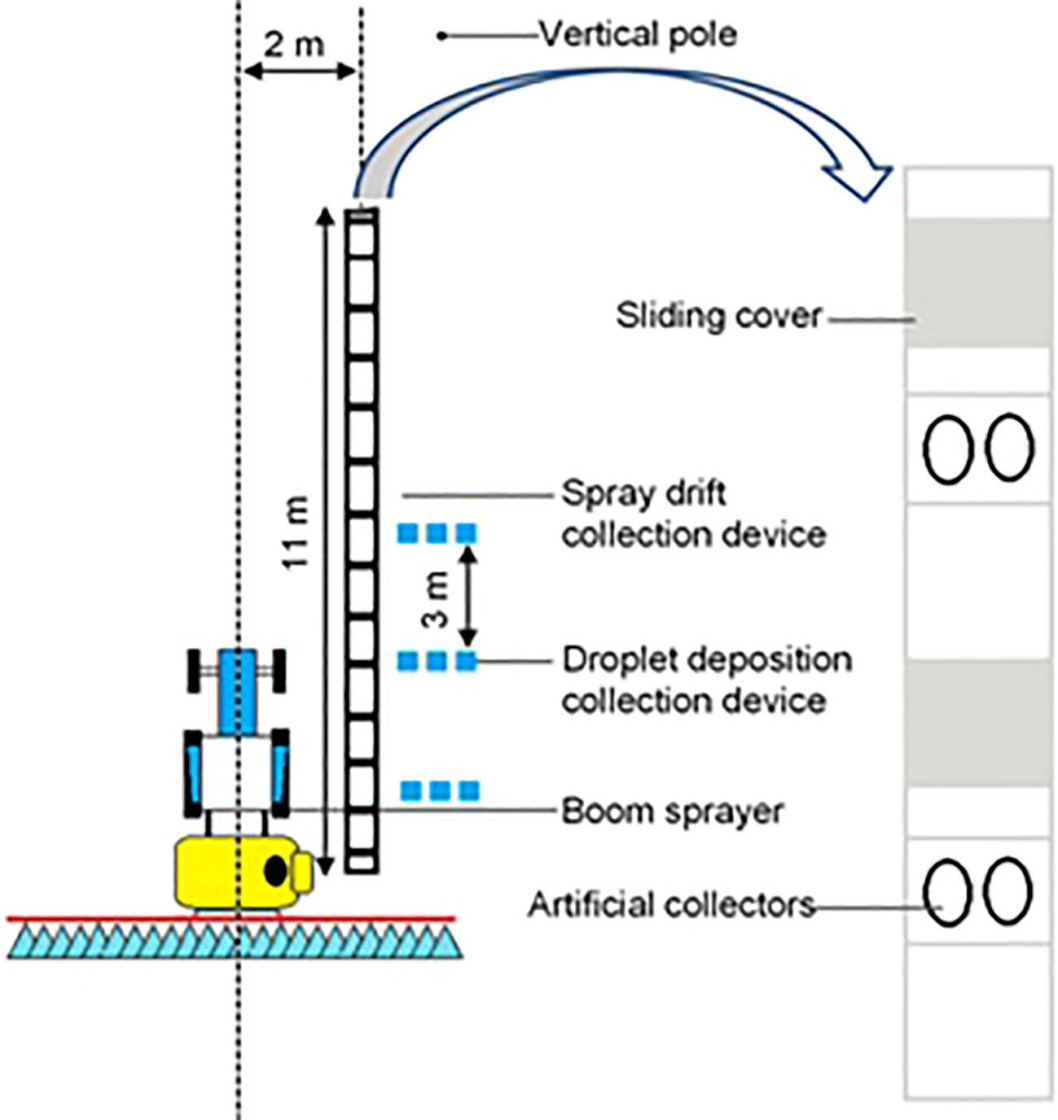
Test bench to assess potential spray drift from horizontal boom sprayers.

The deposition and drift potential of nozzles was determined in accordance with ISO 22401 (2015), with some modifications as done before by different researchers (Balsari et al., 2017; Nuyttens et al., 2017). The drift test bench (**Figure 1**) was manufactured by Advanced Agricultural Measurement System (Belgium). Before each test, the to-be-tested nozzles were mounted on the boom sprayer, and the boom height was adjusted so that the nozzles were 50 cm vertically above the test bench profile. The test bench was located under the center of one side of the boom sprayer, and two petri dishes with a diameter of 9 cm were placed in each groove of the spray drift collection device. The length of the test bench was 11 m. Additionally, three lines of Mylar cards of 100 cm^2^ were horizontally arranged beside the test bench under the spraying swath of the boom sprayer for measuring the spray deposition of the nozzles. After placing the petri dishes and Mylar cards and passing of the sprayer (**Figure 1**), the sliding covers above the droplet test device were closed by the control unit.

Each nozzle–pressure combination was tested three times. All tests were carried in windless conditions. The average temperature during the test period was 23°C to 26°C, and the relative humidity ranged from 50% to 65%. Spray deposition on the samplers was determined using lemon chrome (Shanghai Dyestuffs Research Institute Co., Ltd., China) as a tracer with a mass concentration of 0.5% in the spray solution.

A quantitative volume of deionized water was added into the collecting petri dishes and Mylar cards in the laboratory for elution, and the absorbance of the eluent at 425 nm was measured by a visible spectrophotometer (722s, Shanghai Jinghua). According to ISO 24253-1 and ISO 22401, the calculation formula of spray deposition is shown below:


(1) $$\beta_{dep}orD_{i}=\left[\left(\rho_{smpl}–\rho_{blk}\right)\times V_{dil}\right)]/[\rho_{spray}\times A_{col}]$$

where *β*_dep_ is the spray deposition on the Mylar card (μl/cm^2^), *D_i_* is the spray deposition on the petri dish (μl/cm^2^), *ρ*_smpl_ is the absorbance of the eluent, *ρ*_blk_ is the absorbance of the deionized water, *V*_dil_ is the volume of the added eluent (μl), *ρ*_spray_ is the absorbance of the calibration solution, and *A*_col_ is the collector area (cm^2^).

Drift potential value was obtained by calculating the droplet deposit of the test nozzles at various positions under different spray pressures. Once the amount of tracer on every single collector was determined, the drift potential value calculation process continues according to the following formula:


(2) $$d_{PV}=\sum D_{i}/d_{RS}\times 100$$

where *d*_PV_ is the drift potential value (dimensionless), and *d*_RS_ is the theoretical spray deposition per unit area (μl/cm^2^).

In order to calculate the drift reduction percentage (DRP) of the nozzles, nozzle ST110-03 at 0.3 MPa was taken as a reference, whose DRP was set as 0. The DRP of other nozzle–pressure combinations were calculated according to the following equation:


(3) $$DRP\;=\;(d_{Pvref}-\;d_{PV})/d_{Pvref}\times 100\%$$

where *d*_Pvref_ is the drift potential value of the ST110-03 nozzle at 0.3 MPa.

### 2.4 Field trials

#### 2.4.1 Experimental area

The field experiments were carried out at the field of Miyun County, Beijing, China (geographical longitude 116.83° E, latitude 40.37°N) without surrounding buildings or trees affecting the wind conditions. The field trial was in crop rotations of wheat and maize.

#### 2.4.2 Spray drift

**Figure 2:**
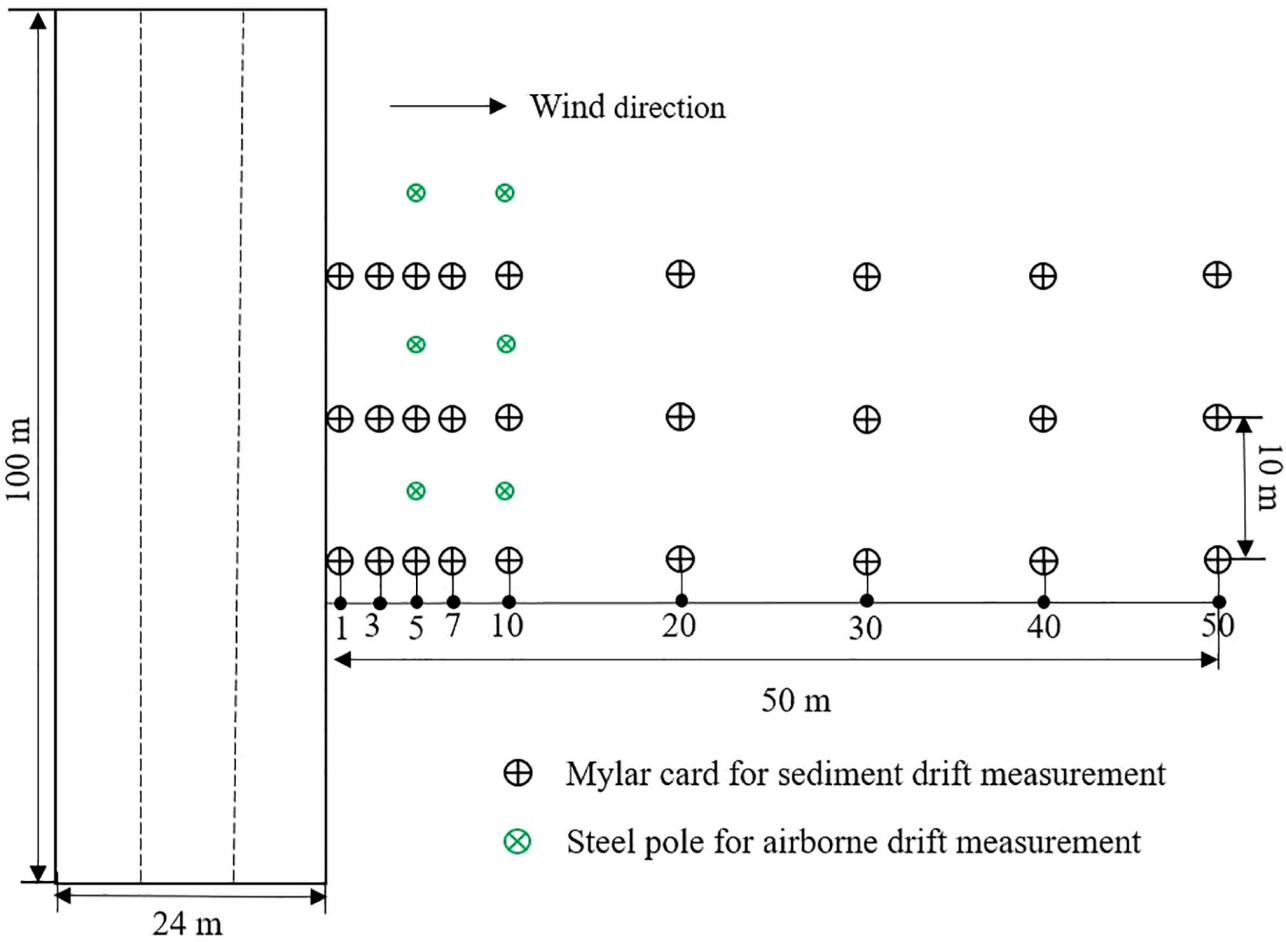
Layout of field sampling for the spray drift trails.

Spray drift was measured in the maize field with the four application techniques mentioned above, *i*.*e*., LU120-03, LU120-05, IDKT120-03, and IDKT120-05 at 4 km/h at 0.3 MPa. The spray liquid was prepared by mixing acetochlor + atrazine + 2,4-D SE with a dilution factor of 250 with water (acetochlor of 340 g a.i.L^-1^, atrazine of 280 g a.i.L^-1^, and 2,4-D of 80 g a.i.L^-1^). The spray drift of the nozzles was determined in accordance with ISO 22866 (2005). Each application technique sprayed three swaths (24 m in width), and the boundary of the last spray swath of the boom sprayer was set as the edge of the directly sprayed area. The traveling direction of the boom sprayer was perpendicular ( ± 15°) to the wind direction. The determination of spray drift included both sediment drift and airborne drift (**Figure 2**).

For each replication, sediment drift was monitored using three sampling lines of Mylar cards (50 mm × 100 mm) positioned downwind of the sprayed zone on the ground. The distance between each sampling line was 10 m. In each sampling line, the Mylar cards were arranged at 1, 3, 5, 7, 10, 20, 30, 40, and 50 m downwind of the sprayed area. For airborne drift, three steel poles were positioned at 5 and 10 m downwind of the sprayed area with a distance of 10 m between each pole. The Mylar cards were fixed on each pole at heights of 1, 2, 3, 4, 5, and 6 m. After the pesticide application, each Mylar card was removed and placed in a separate sealed bag and then stored at -20°C until analyzed by chromatography.

The chromatographic system was a Thermo U3000 series HPLC equipped with a reverse-phase column, and atrazine was used as the tracer to quantify the spray drift. The temperature of the column was kept at 30°C. The injection volume was 20 μl. The mobile phase was composed of acetonitrile and 0.1% aqueous formic acid (70/30, *v/v*) at a flow rate of 0.3 ml/min. The Mylar cards were extracted with 10 ml acetonitrile in the sealed bag. One milliliter of the upper solution was filtered through a 0.22-μm syringe filter and placed into an auto-sampler vial for HPLC analysis. The detection wavelength was 220 nm, and the retention time of atrazine was 3.43 min. Linearity was studied in the range of 0.01–0.5 mg/L for atrazine with five calibration points (0.01, 0.05, 0.1, 0.2, and 0.5 mg/L) by matrix-matched standard calibration in blank extracts of Mylar card. The calibration curves showed a good linearity with a correlation coefficient (*R* ^2^) of 0.9987.

A weather station ZENO-3200 (Pri-eco Company Limited, USA) was used to record the wind speed, and Testo 350-XL (Testo SE and Co. KGaA, Germany) was used to record the temperature and humidity during the field experiments. These meteorological stations were located upwind of the field at a height of 2 m. During field trials, the wind speed ranged from 1.1 to 3.2 m/s, the average wind speed was 1.7 to 1.8 m/s, the relative humidity ranged from 36.3% to 47.4%, and the temperature was between 28.1°C and 30.4°C. The details of the meteorological conditions of the nozzles during the trial are shown in **Table 2**.

**Table 2:** Meteorological conditions during the field trial.

Nozzle type	Minimum wind velocity (m/s)	Maximum wind velocity (m/s)	Average wind velocity (m/s)	Relative humidity (%)	Temperature (°C)
LU120-03	1.5	2.9	1.8	38.5	28.9
LU120-05	1.5	2.8	1.8	41.0	28.1
IDKT120-03	1.4	2.8	1.7	36.3	30.4
IDKT120-05	1.1	3.2	1.7	47.4	29.5

#### 2.4.3 Weed control

In maize, weed control treatments were performed in the same field as the spray drift trials. The broad-leaf weeds in the maize field mainly included *Commeline communis*, *Chenopodium album*, *Amaranthus retroflexus*, *Pharbitis nil Choisy*, *Descurainia sophia*, and *Abutilon theophrasti Medic*, and the gramineous weeds included carbgrass (*Digitaria ciliaris*), goosegrass herb (*Eleusine indica*), and barnyardgrass [*Echinochloa crusgali* (Linn.) *Beauv.* var. *praticola Ohwi*].

In wheat, the pesticides used were commercial formulations of Tribenuron-methyl WG (containing 750 g a.i.L^-1^) and 2,4-D EC (containing 570 g a.i.L^-1^). The application rates of Tribenuron-methyl and 2,4-D were 15 and 500 g/ha (the recommended dosages), respectively. The main weeds in the wheat field are *Capsella bursa-pastoris*, *Descurainia sophia*, and wild oat (*Avena fatua* L.).

For both maize and wheat, the weed species and the numbers in each plot were determined prior to the herbicide application. The weed control efficacy was determined by counting the number of weeds at 20 (maize) and 30 days (maize and wheat) after spraying. In addition, the fresh weight inhibition rates after 30 days of herbicide application in the maize field were also investigated. For both the maize and wheat fields, there were five sampling points selected in each plot for efficacy evaluation, and the area of each sampling point was 1.0 m × 1.0 m.

### 2.5 Statistical analysis

The normalized volumes of each petri dish and Mylar card and the control efficacy of weeds were analyzed with a factorial analysis of variance. The significance of the differences in the spray deposition, drift potential, and the control efficacy of weeds between different treatments was evaluated by Duncan’s test for a significance level of 95%. All the analyses were performed with the statistical software SPSS v.19.0 (SPSS Inc., Chicago, IL, USA).

## 3 Results and discussion

### 3.1 Droplet spectrum and classification

**Table 3** shows the results of VMD and *V*_100_ of the droplet size spectra of each tested nozzle–pressure combination. Increasing the nozzle size increased the nozzle flow rate (**Table 1**) and, in most cases, also the droplet sizes. Thus, the droplet size spectra of the ISO 03 ST, LU, and IDK nozzles were all smaller than their corresponding ISO 05 nozzle sizes for the same spray pressure. However, the twin air-induction flat-fan nozzle IDKT of ISO 03 showed significantly higher VMD values (546.6, 415.8, and 355.0 μm at 0.2, 0.3, and 0.4 MPa, respectively) compared with the ISO 05 IDKT with VMD values of 395.4, 329.1, and 287.9 μm at the same pressure levels.

**Table 3:** Volume percentage of droplets smaller than 100 μm (%, *V*_100_), volume median diameter (μm, VMD), and droplet size classification (DC) for different nozzle–pressure combinations.

Nozzle type	0.2 MPa			0.3 MPa			0.4 MPa		
	*V*_100_	VMD	DC	*V*_100_	VMD	DC	*V*_100_	VMD	DC
ST110-03	15.70	171.4	F	22.09	151.9	F	25.80	142.8	VF
LU120-03	16.89	173.8	F	23.38	154.1	F	26.46	144.3	VF
IDK120-03	2.58	363.7	M	4.35	307.2	M	5.28	267.2	M
IDKT120-03	1.39	546.6	VC	2.30	415.8	C	2.77	355.0	C
ST110-05	9.92	208.9	F	14.52	186.7	F	18.94	167.3	F
LU120-05	12.60	195.1	F	17.93	174.2	F	19.95	168.4	F
IDK120-05	1.34	404.6	C	1.95	361.9	C	2.40	319.0	M
IDKT120-05	2.00	395.4	C	2.45	329.1	M	2.94	287.9	M

VF, very fine; F, fine; M, medium; C, coarse; VC, very coarse.

For the same nozzle, the higher the spray pressure, the smaller the droplet size. ISO 25358 (2018) provides a reference system for defining and classifying the droplet size spectra. According to this classification, in the range from 0.2 to 0.4 MPa, the droplet size classes of the LU and ST nozzles were very similar and ranged from fine to very fine, while the droplets of the air-induction nozzles IDK and IDKT were medium or coarse depending on the nozzle size and pressure.

The droplet size is an important indicator to evaluate the characteristics of atomization and to compare the atomization quality of different nozzles (Cunha et al., 2019). It can be used to determine the deposition distribution of the pesticide droplets on the target to a certain extent, and it is also an important parameter to predict the drift potential of the nozzles. The ISO standard 25358 states that the smaller the droplet size is, the more beneficial for the droplets to be attached on the target. Small droplets, however, increase the drift potential. The droplet size spectra of the air-induction nozzles were coarser than that of the standard flat-fan nozzles at the same spray conditions. This was because the solution sprayed from the air-induction nozzle is mixed with air entering through the air-induction holes to produce droplets containing a large number of air bubbles, resulting in a large droplet size for the same flow rate.

### 3.2 Deposition and drift potential of nozzles

**Table 4** shows the spray deposition of ISO 03 and 05 nozzles under different spray pressures. For the ISO 03 nozzles, when the spray pressure was 0.2 MPa, the deposition of the four series of nozzles was in the range from 1.34 to 1.39 μl/cm^2^, and there were no significant differences (*α* = 0.05). However, the deposition of air-induction nozzles was higher than that of standard flat-fan nozzles at the pressure levels of 0.3 and 0.4 MPa. Especially the deposition of twin flat-fan air-induction IDKT120-03 nozzles was significantly higher than that of flat-fan nozzles. The deposition of the various types of ISO 05 nozzles confirms this positive effect of air-induction nozzles on spray deposition.

**Table 4:** Spray deposition (μl cm^-2^) of ISO 03 and 05 standard flat-fan nozzles (ST and LU) and air-induction nozzles (IDK and IDKT) at different spray pressures.

Nozzle series	ISO 03			ISO 05		
	0.2 MPa	0.3 MPa	0.4 MPa	0.2 MPa	0.3 MPa	0.4 MPa
ST110	1.34a	1.57b	1.74c	1.73c	2.21b	2.50b
LU120	1.35a	1.67ab	1.85bc	1.78bc	2.32b	2.53b
IDK120	1.37a	1.72a	1.95ab	1.90b	2.36b	2.82a
IDKT120	1.39a	1.76a	2.00a	2.26a	2.51a	2.90a

The results in the table are averages of three replicates. Different letters in the same column indicate significant difference at P<0.05 level.

On the whole, in terms of increasing the amount of deposition, twin flat-fan air-induction nozzles (IDKT series) were better than single flat-fan air-induction nozzles (IDK series), and air-induction nozzles were better than standard flat-fan nozzles. The increase of the spray deposition of air-induction nozzles, especially the twin flat-fan air-induction nozzles, is linked with their higher drift reducing potential.

**Figure 3:**
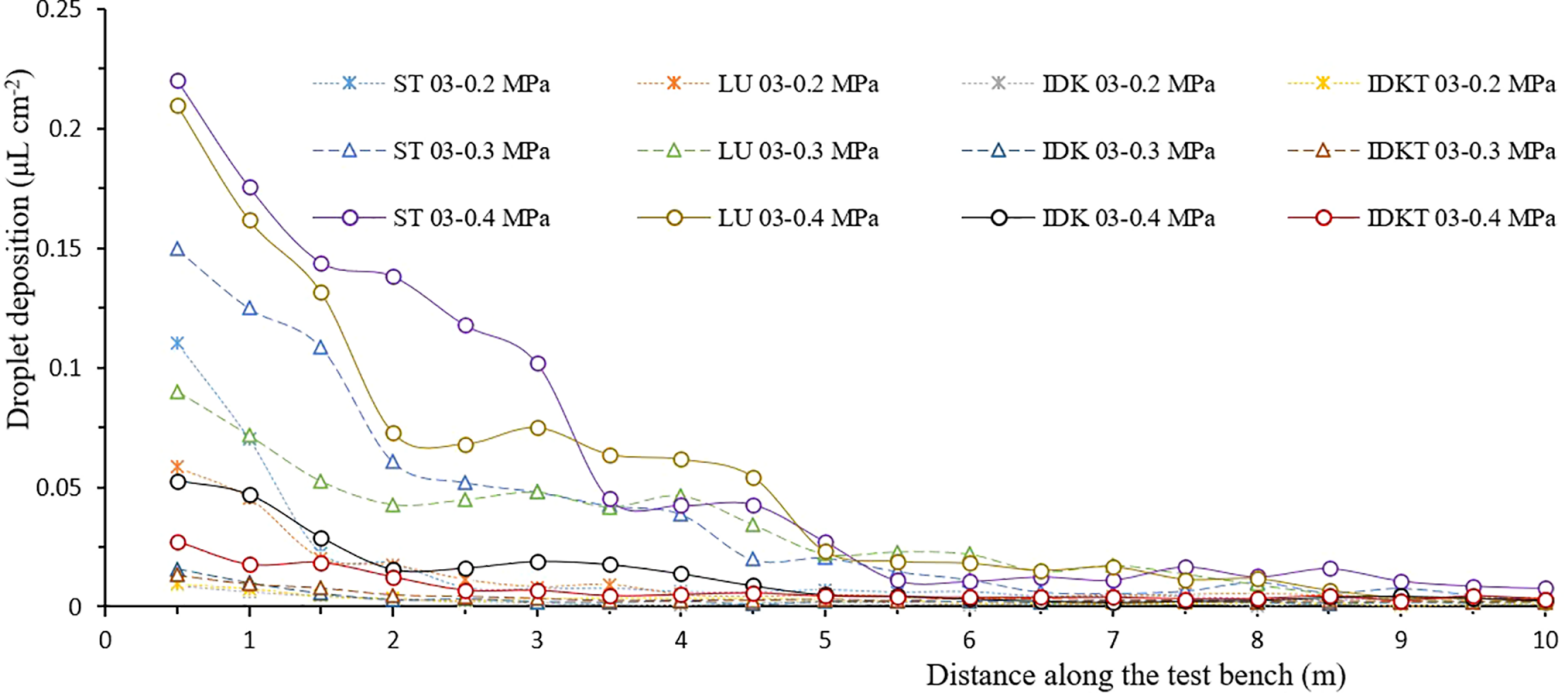
Spray deposit profiles of different ISO 03 nozzle-pressure combinations along the test bench.

**Figure 4:**
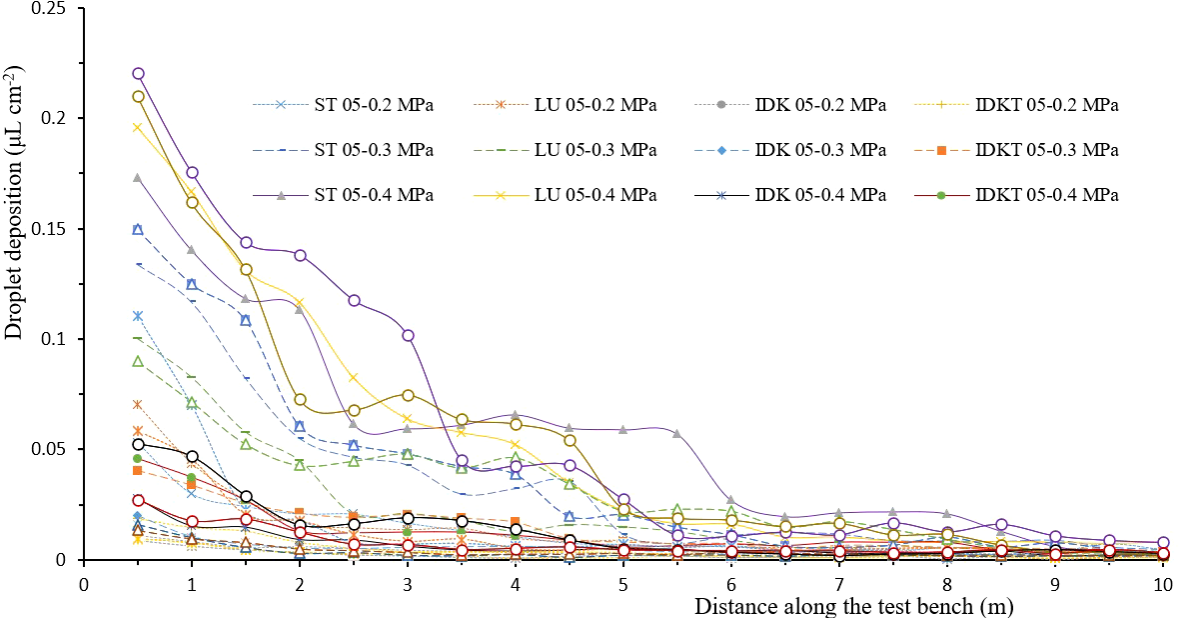
Spray deposit profiles of different ISO 05 nozzle-pressure combinations along the test bench.

**Figures 3**, **4** show the spray deposit profiles collected by petri dishes at different distances on the drift test bench. These curves indicated that the spray deposit of each nozzle decreased with the increase of the collection distance, and the greatest part of spray deposition was located on the first 5 m of the bench located closest to the actuator pole.

**Table 5** shows the drift potential values and drift reduction percentages for all nozzle–pressure combinations. The *d*_PV_ of nozzles increased with an increase of spray pressure—for example, the DRP values of ST110-03 nozzle were 43.3%, 0% (reference), and -50.0% at spray pressures of 0.2, 0.3, and 0.4 MPa, respectively. Not surprisingly and because of their similar droplet size characteristics, very similar DRP values were observed for the other conventional flat-fan nozzle LU120-03, with DRP values of 52.8%, 2.6%, and -55.7% at the same pressure.

**Table 5:** Drift potential value and drift reduction percentage (%) of the different nozzle–pressure combinations.

Nozzle type	Drift potential value (-)			Drift reduction percentage (%)		
	0.2 MPa	0.3 MPa	0.4 MPa	0.2 MPa	0.3 MPa	0.4 MPa
ST110-03	23.7a	41.8a	62.7a	43.3	0.0	-50.0
LU120-03	19.7a	40.7a	65.1a	52.8	2.6	-55.7
IDK120-03	4.0b	8.3b	16.2b	90.4	80.1	61.2
IDKT120-03	6.2b	7.7b	12.7b	85.2	81.6	69.6
ST110-05	14.7a	29.8a	45.4a	64.8	28.7	-8.6
LU120-05	18.7a	27.1a	48.5a	55.3	35.2	-16.0
IDK120-05	5.4b	6.1c	8.1b	87.1	85.4	80.6
IDKT120-05	7.5b	12.1b	10.7b	82.1	71.0	74.4

Different letters in the same column indicated significantly different at P<0.05 level.

In all cases, the DRP values obtained with all the air-induction nozzles were greater than that of standard flat-fan nozzles. The highest DRP was achieved for the IDK120-03 nozzle at 0.2 MPa with a percentage of 90.4%. Among all these air-induction nozzle–pressure combinations, the drift reduction percentage of IDK 120-03 nozzle sprayed at 0.4 MPa was the lowest but still above 60%. This was predominantly caused by an increase in the percentage of *V*_100_ (**Table 3**) due to the increased spray pressure. Smaller droplets settle down more slowly and are more influenced by environmental factors (*e*.*g*., wind) compared with larger droplets. Compared with the standard flat-fan nozzles, the spray pressure had a minor effect on the medium and coarse droplet sprays of the IDK and IDKT nozzles.

The drift potential trends of the four ISO 05 nozzles were consistent with those observed for the ISO 03 nozzles. Under the same spray pressure, the DRP of ST110-05, LU120-05, and IDK120-05 nozzles were greater than that of the same types of ISO 03 nozzles, while the DRP of the IDKT120-05 was lower than that of IDKT120-03. This result is fully in line with the result of the droplet size test (**Table 3**), which confirms the effect of droplet size on drift potential.

**Figure 5:**
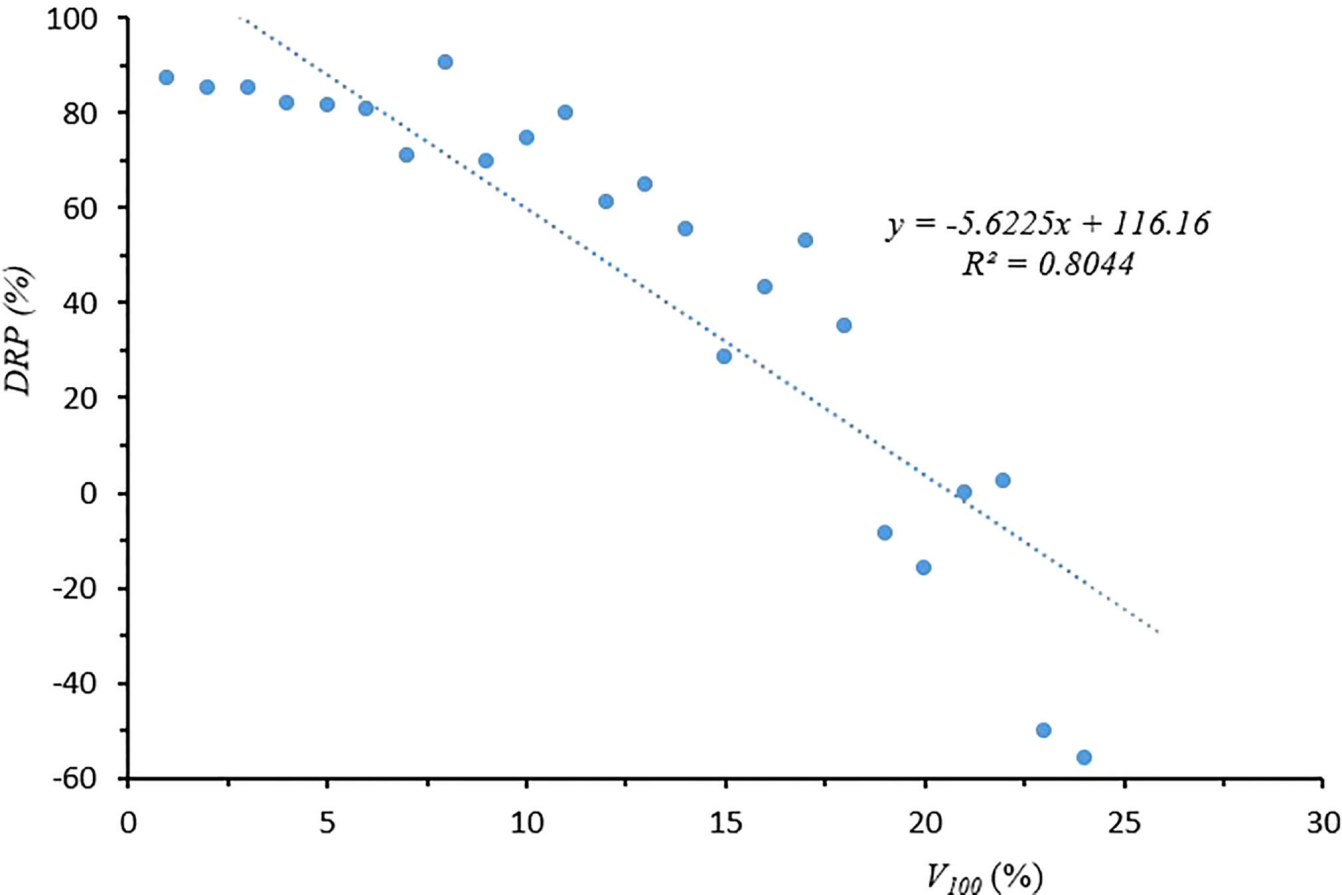
Correlation between droplet size characteristics V100 and drift reduction percentage (DRP).

**Figure 5** shows the simple first-order linear regression between DRP (%) and *V*_100_ (%): DRP = -5.6225 *V*_100_ + 116.16, with an *R* ^2^ of 0.80. From these results, it is clear that droplet size characteristics, mainly *V*_100_, is strongly linked with DRP. Other researchers also considered droplets smaller than 100 or 200 μm to be the most drift prone (Bouse et al., 1990; Nuyttens et al., 2007).

Furthermore, deposition and drift of droplets are also related to their settling velocity. Studies have shown that the droplet initial velocity of standard flat-fan nozzles is significantly higher than that of air-induction nozzles (Wang et al., 2015). However, as these atomized high-velocity droplets travel away from the standard flat-fan nozzle, their settling velocity decreased dramatically because of their small size. In contrast, the initial setting velocity of the bigger droplets atomized by the air-induction nozzles can be maintained for a longer distance due to the large inertia, which is beneficial to increase deposition and reduce drift.

### 3.3 Sediment and airborne spray drift in the field

**Figure 6:**
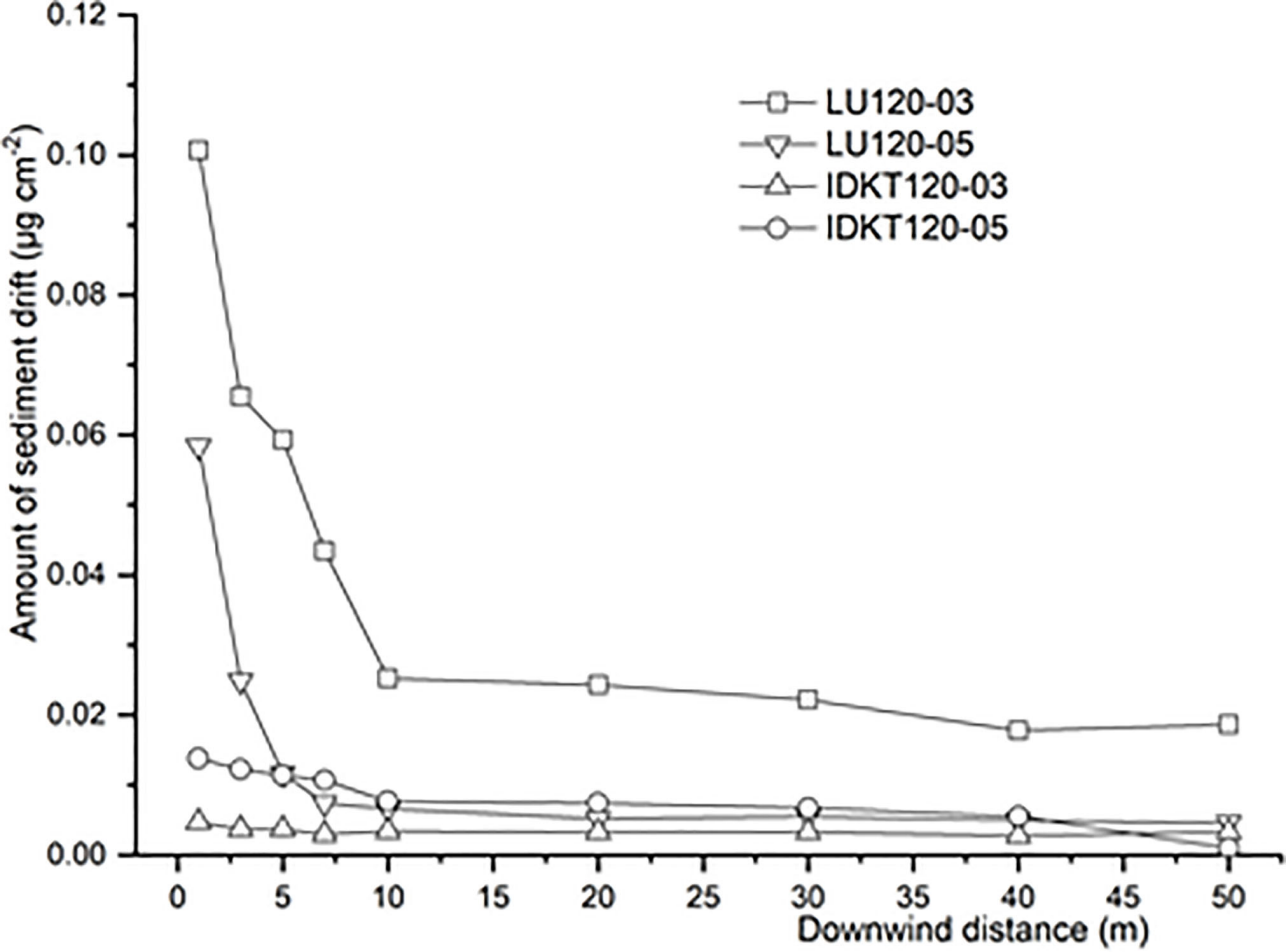
Sediment drifts of nozzles in different downwind distances.

**Figure 6** shows the sediment spray drift at different downwind distances. The spray drift of LU120-03 nozzle was higher than that of the other three nozzles, which is in accordance with the drift potential assessed with the test bench and the droplet size characteristics. In general, the sediment drift of nozzles decreased when the downwind distance increased, and this tendency is most pronounced for the flat-fan nozzles. The sediment spray drift values of standard flat-fan nozzles are clearly higher than those of air-induction nozzles. The sediment drift of LU120-03 and LU120-05 nozzles at 1 m downwind was 0.100 and 0.058 μg/cm^2^, respectively. However, the sediment drift of IDK120-03 and IDK120-05 nozzles was no more than 0.01 μg/cm^2^. Compared with standard flat-fan nozzles, the air-induction nozzles could effectively reduce the sediment drift for herbicides application in the field.

**Figure 7:**
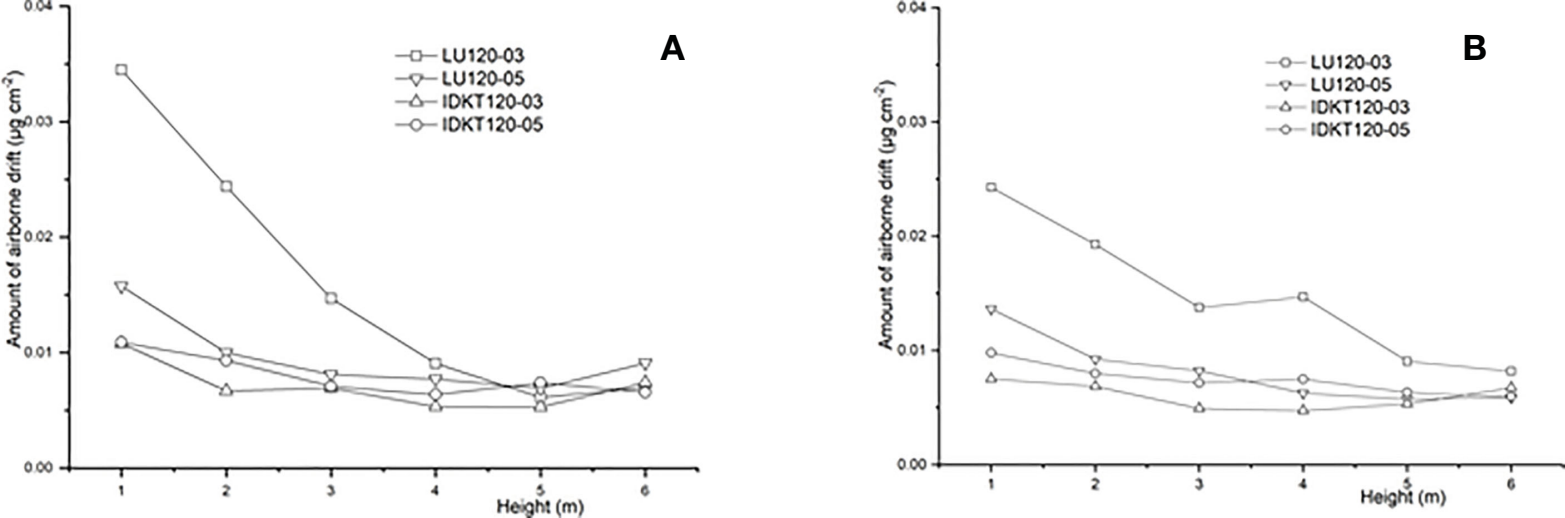
Airborne drifts of nozzles at 5 m **(A)** and 10 m **(B)** downwind distances.

The airborne spray drift results of flat-fan nozzles and air-induction nozzles at different heights are shown in **Figure 7**. The amount of airborne spray drift pesticide (μg/cm^2^) was calculated from the average value of the Mylar cards of the same height. The results showed that the airborne drift of nozzles decreased with the increase of the height within the downwind distance from 5 to 10 m. Airborne drift was mainly concentrated below 4 m, and the airborne drift values of flat-fan nozzles were larger than that from the air-induction nozzles. At the downwind distance of 5 m, the airborne drift values of LU120-03, LU120-05, IDK120-03, and IDK120-05 were 0.034, 0.016, 0.011, and 0.011 μg/cm^2^, respectively. In the same way, at the downwind distance of 10 m, the airborne drift values of LU120-03, LU120-05, IDK120-03, and IDK120-05 were 0.024, 0.014, 0.008, and 0.009 μg/cm^2^, respectively. Droplets below 100 μm contribute significantly to drift losses (Nuyttens et al., 2017), and the smaller the spray droplet was, the longer it remains airborne and the higher the possibility for it to be carried away by a crosswind (Wang XN et al., 2018). The *V*_100_ of the LU120-03 nozzle was 22.09%, which was higher than 17.93% of the LU1120-05 nozzle (**Table 3**). The *V*_100_ of IDK120-03 and IDK120-05 nozzles was 4.35% and 1.95%, respectively, which were significantly lower than those of the two flat-fan nozzles. The overall consistency between drift potential, sediment drift, and airborne drift confirms the great drift reducing potential of air-induction nozzles.

### 3.4 Herbicide efficacy

#### 3.4.1 In the maize field

After 7 days of herbicide application, the growth status of the weeds in the maize field was investigated, and it was found that their growth points show symptoms of chlorosis; some weeds showed slight deformities, and the leaf edges turned yellow. **Table 6** shows the biological efficacy of the different nozzles at 20 and 30 days after herbicide application. At 20 days after herbicide application, the control efficacy on weed density was more than 85% for all tested nozzles, and there were no significant differences between standard flat-fan nozzles and air-induction nozzles. Compared with 20 days after application, the weed control efficacy of 30 days after application was decreased. Nonetheless, the control efficacy values were still more than 80%, without significant differences between flat-fan nozzles and air-induction nozzles.

**Table 6:** Weed plant number before treatment (WPNBT), control efficacy (%) on weed plant number, and fresh weight of weeds in the maize field using standard flat-fan nozzles and air-induction nozzles.

Nozzle	WPNBT	On weeds plant number				On fresh weight	
		20 DAT		30 DAT		30 DAT	
		Weeds	Efficacy	Weeds	Efficacy	Weight	Efficacy
	m^-2^	m^-2^	%	m^-2^	%	g	%
LU120-03	76.5	11.0	90.9a	15.2	82.9a	3.2	92.3b
LU120-05	95.0	13.5	85.0a	18.0	83.1a	5.3	87.3c
IDKT120-03	62.0	9.5	86.3a	13.5	80.5a	1.9	95.4a
IDKT120-05	119.2	13.2	90.1a	19.5	85.4a	5.0	88.0c
Control	109.0	122.0	–	127.0	–	41.6	–

DAT, days after treatment. Different letters in the same column indicated significantly different at P<0.05 level.

Based on the fresh weight of the weeds 30 days after herbicide application (**Table 6**), the IDKT120-03 nozzle had the best control efficacy of 95.4%, followed by LU120-03 nozzle with 92.3%, while the control efficacy of the LU120-05 and IDKT120-05 nozzles was 87.3% and 88.0%, respectively. The control efficacy based on the fresh weight of the weeds of air-induction nozzle IDKT120-03 was significantly higher than that of the other three nozzle types. In addition, the control efficacy of the flat-fan nozzle LU120-03 was significantly higher than both ISO 05 nozzles (LU120-05 and IDKT120-05). LU120-03 nozzle had the finest droplet size spectra, leading to a higher chance of hitting small weed target and a higher droplet density on the target. Therefore, its control efficacy was better than that of LU120-05 and IDKT120-05 nozzles.

#### 3.4.2 In the wheat field

After 14 days of herbicide application in the wheat field, the growth status of the wheat was investigated, and it was found that the wheat grew well and there was no phytotoxicity for all tested nozzles. The weeds grew slowly with yellow leaves, and some plants were withered, but the roots were not dead yet at 14 days after treatment. At 30 days after application, the plant density of the weeds in each plot was investigated, and the control efficacy based on the number of weeds for each nozzle was calculated.

**Figure 8:**
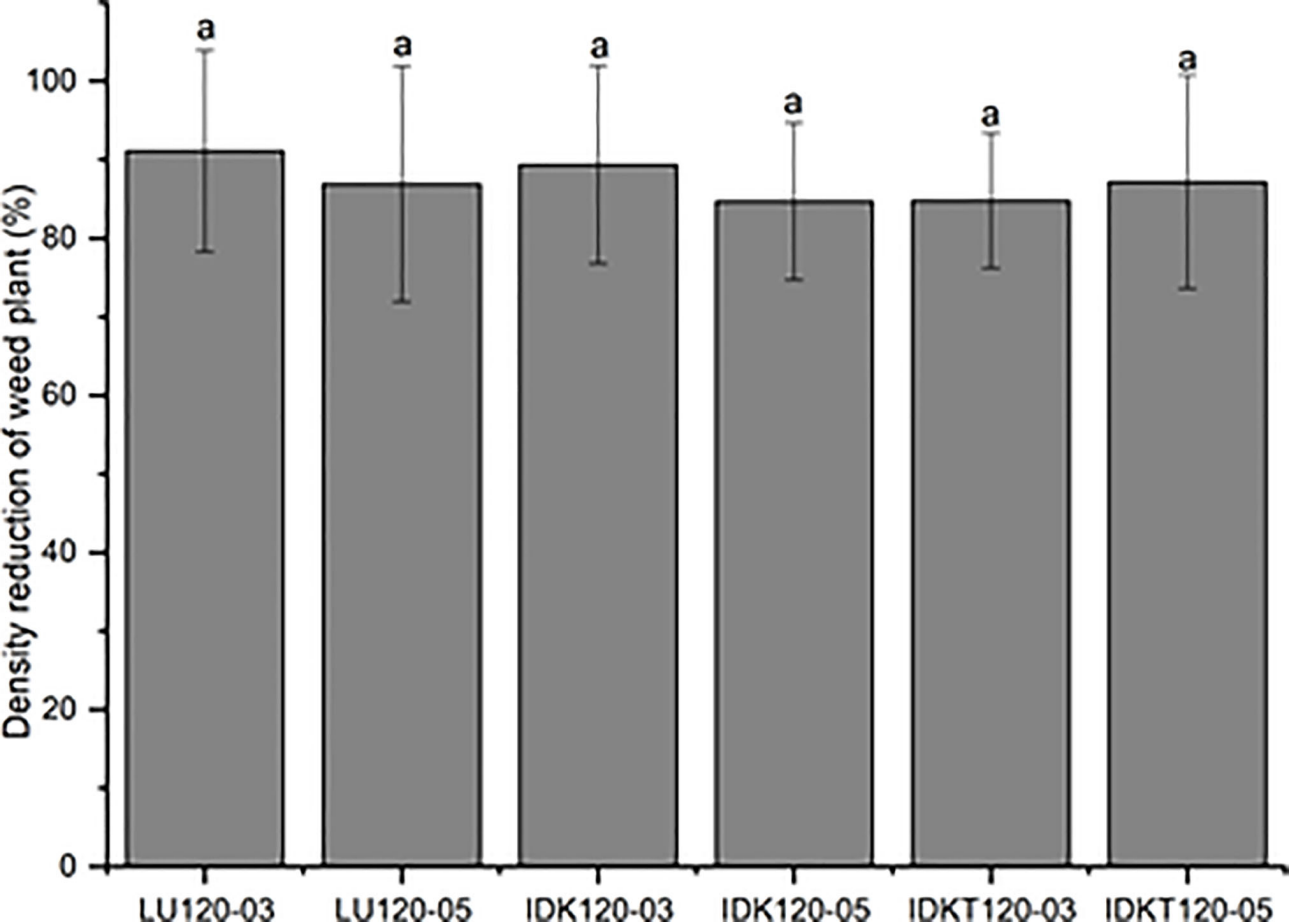
Biological efficacy of herbicides applied in wheat field using standard flat fan nozzles and air-injector nozzles.

**Figure 8** shows the results in terms of the percentage reduction of the number of weed plants in the wheat field. The control efficacy of standard flat-fan nozzles LU120-03 and LU120-05 was 91.1% and 86.9%, respectively. Similarly, the control efficacy of air-induction nozzles IDK120-03 and IDK120-05 was 89.4% and 84.7% respectively, and the control efficacy of twin flat-fan air-induction nozzles IDKT120-03 and IDKT120-05 was 84.8% and 87.2%, respectively.

Comparing the biological efficacy of standard flat-fan nozzles and air-induction nozzles, the control effects of the six tested nozzles were all more than 84%, and there were no significant differences among various nozzles. The results may be explained by a combination of factors, such as the droplet characteristics, meteorological conditions, and the distribution of weeds in the field. Although the fine droplets of standard flat-fan nozzles generally improve adhesion and droplet density on the weed targets, the fine droplets also have a greater drift potential, which might reduce the deposition on the targets. On the contrary, the coarse droplets of air-induction nozzles have a lower drift potential, and these coarse droplets are more prone to rebound or shatter on the target leaf surface (Zwertvaegher et al., 2014; Delele et al., 2016; Franca et al., 2020). In addition, the variation in meteorological conditions and the distribution of weeds in the field increased the deviation between sampling points. These results indicate that air-induction nozzles IDK and IDKT had the same control efficacy as that of standard fan nozzles for herbicide application in the field.

## 4 Conclusions

The droplet size measurement showed that the droplet size classes of the standard flat-fan LU and ST nozzles were very similar and ranged from fine to very fine, while the droplet size classes of the air-induction nozzles IDK and IDKT were medium or coarse depending on the spray pressure and nozzle size. Drift potential trials using a drift test bench showed that droplet size characteristics, mainly *V*_100_, are strongly linked with drift reduction potential. Both drift potential and field drift trials showed that the compact air-induction nozzles (IDK and IDKT) significantly reduced the spray drift compared with standard flat-fan nozzles. This is of great significance for the protection of the environment and the surrounding sensitive crops. In terms of weed control biological efficacy, there were no significant differences between standard flat-fan nozzles and air-induction nozzles. In all cases, the efficacy values were above 80% both in maize and in wheat. Therefore, it is recommended to use air-induction nozzles when spraying herbicides in the field.

## Data availability statement

The original contributions presented in the study are included in the article/supplementary material. Further inquiries can be directed to the corresponding author.

## Author contributions

Conceptualization, YL; data curation, SW and XiL; formal analysis, SW and LZ; investigation and methodology, SW, XiL, and LZ; Supervision, YL and XuL; writing—original draft, SW and XuL; writing—review and editing, DN and YL. All authors contributed to the article and approved the submitted version.
